# Cabergoline-Induced Hypoprolactinemia May Attenuate Cardiometabolic Effects of Atorvastatin: A Pilot Study

**DOI:** 10.1159/000527333

**Published:** 2022-10-04

**Authors:** Robert Krysiak, Karolina Kowalcze, Bogusław Okopień

**Affiliations:** ^a^Department of Internal Medicine and Clinical Pharmacology, Medical University of Silesia, Katowice, Poland; ^b^Department of Pediatrics in Bytom, School of Health Sciences in Katowice, Medical University of Silesia, Bytom, Poland

**Keywords:** Cardiometabolic risk factors, Dopamine agonists, Insulin sensitivity, Lipid profile, Prolactin deficiency, Statins

## Abstract

**Introduction:**

Hypoprolactinemia, which is usually a consequence of treatment with inadequate high doses of dopaminergic agents, is a poorly understood clinical condition. The aim of the current study was to investigate whether the cardiometabolic effects of statin therapy differ between patients with low prolactin production and patients with normal levels of this hormone.

**Methods:**

We studied two groups of cabergoline-treated premenopausal women with hypercholesterolemia matched for age, plasma lipids, cabergoline dose, and treatment duration: 11 women with hypoprolactinemia (group A) and 15 women with plasma levels of this hormone within the reference range (group B). The control group (C) included 25 dopaminergic-naïve normoprolactinemic women, matched for age and lipid levels. Plasma lipids, insulin sensitivity, and levels of uric acid, high-sensitivity C-reactive protein (hsCRP), fibrinogen, homocysteine, and 25-hydroxyvitamin D were measured before and after 14-week treatment with atorvastatin (20 mg daily).

**Results:**

Patients with hypoprolactinemia were more insulin-resistant, had lower values of total testosterone and free androgen index, and had higher levels of hsCRP and fibrinogen than individuals with normal prolactin levels. Although atorvastatin reduced total and LDL cholesterol and hsCRP in all study groups, this effect was stronger in groups B and C than in group A. Only in groups B and C, the drug decreased uric acid, fibrinogen, and homocysteine and increased 25-hydroxyvitamin D. In turn, only in group A, atorvastatin worsened insulin sensitivity and reduced free androgen index.

**Conclusion:**

Coexisting hypoprolactinemia may have an unfavorable impact on the pleiotropic effects of statins.

## Introduction

Apart from endocrine manifestations, long-lasting hyperprolactinemia is associated with many negative metabolic outcomes, such as decreased insulin sensitivity, prediabetes, atherogenic dyslipidemia, excessive fat accumulation resulting in overweight and obesity, endothelial dysfunction, as well as premature atherosclerotic lesions in the arterial wall [1, 2, 3, 4, 5]. These effects are mitigated or reversed if patients receive dopaminergic drugs [6, 7]. Treatment with high doses of these agents, particularly with cabergoline, may, however, lead to the development of hypoprolactinemia, which is usually asymptomatic [8]. The only evident clinical manifestation of this condition in humans is the absence of lactation after delivery [9]. However, Corona et al. [10] showed that prolactin concentrations in the lowest quartile in males with sexual dysfunction were associated with a higher prevalence of metabolic syndrome. Moreover, Krysiak et al. [11] observed that iatrogenic deficiency of this hormone in young women is associated with reduced production of testosterone, which is a hormone that plays an important role in human metabolism [12]. Finally, despite treatment with glucocorticoids, levothyroxine, and sometimes also recombinant growth hormone, patients (particularly women) with hypopituitarism are characterized by excess mortality, which is mainly attributable to cardiovascular and respiratory diseases [13]. These findings may suggest that prolactin failure predisposes to metabolic disorders, is a risk factor for future cardiovascular events and requires better understanding.

In recent years, it has been found that elevated levels of monomeric prolactin (hyperprolactinemia) [14] and excess of high molecular weight prolactin (macroprolactinemia) [15] attenuate the pleiotropic effects of atorvastatin. These findings suggest that changes in production, processing, and/or metabolism of prolactin may modulate the extra-lipid effects of this agent. To better understand the clinical significance of hypoprolactinemia and the association between circulating prolactin levels and the action of 3-hydroxy-3-methylglutaryl coenzyme A (HMG-CoA) reductase inhibitors, the present study has investigated whether the cardiometabolic effects of atorvastatin differ between individuals with low and normal prolactin production.

## Materials and Methods

### Study Population

**Fig. 1:**
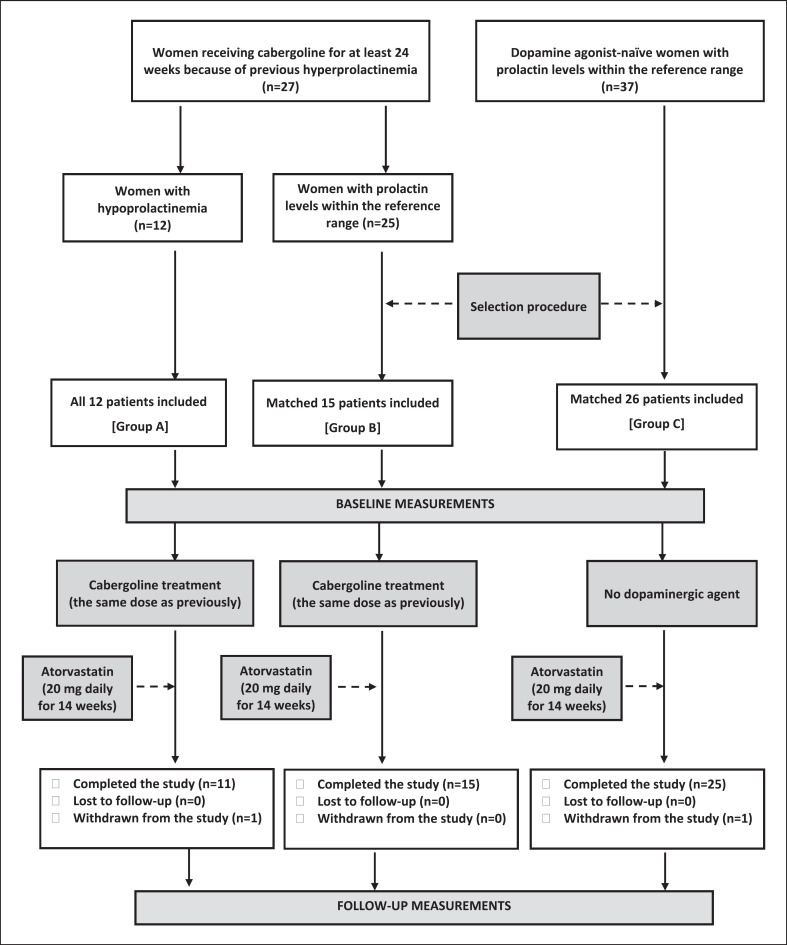
A diagram presenting the flow of patients in the study.

The study population, consisting of 3 groups of patients, was selected among statin-naïve women (aged between 18 and 50 years) with hypercholesterolemia, defined as total cholesterol >190 mg/dL and LDL cholesterol >115 mg/dL. The patients were included if they had a 5% or greater risk of developing a cardiovascular event within the next 10 years and the presence of risk-enhancing factors (family history of premature atherosclerotic cardiovascular disease, premature menopause, history of hypertensive disorders of pregnancy, persistently elevated LDL cholesterol ≥160 mg/dL; metabolic syndrome; ethnicity factors; persistently elevated triglycerides ≥175 mg/mL; and high-sensitivity C-reactive protein (hsCRP) >2.0 mg/L). The 10-year risk for atherosclerotic cardiovascular disease, defined as coronary death or nonfatal myocardial infarction, or fatal or nonfatal stroke, was calculated using the online ASCVD Risk Estimator Plus (http://tools.acc.org/ASCVD-Risk-Estimator-Plus/#!/calculate/estimate/). For women younger than 40 years at presentation, an age of 40 was assigned as the pooled cohort equations for estimation of the 10-year cardiovascular risk are only applicable to individuals aged between 40 and 79 years. Because of previous prolactin excess, groups A and B had been treated with cabergoline for at least 24 weeks. Group A included 12 women with hypoprolactinemia, while group B included 15 women with prolactin levels within the reference range. Hypoprolactinemia was diagnosed if plasma prolactin levels were below 5.0 ng/mL, while the prolactin reference range was defined as 5.0–28.0 ng/mL. Individuals with prolactin concentrations above the reference range were excluded. Group C included 26 normoprolactinemic women never treated with cabergoline or with the remaining dopaminergic drugs. In order to match study groups for age and plasma lipids and groups A and B also for cabergoline dose and treatment duration, groups B and C were selected among a larger number of eligible participants (25 and 37, respectively). Group A included all patients fulfilling the inclusion and exclusion criteria. The selection procedure was performed using the freely available PEPI-for-Windows computer program. In order to limit the effect of seasonal variations in the outcome variables and seasonal confounds, similar numbers of patients were included in the summer and winter months (26 and 27, respectively). The flow of patients through the study is depicted in Figure 1*.*

The exclusion criteria were as follows: cardiovascular disease, endocrine disorders, diabetes, serious diseases affecting prognosis, such as renal failure, hepatic failure, anemia or oncological diseases, inflammatory or autoimmune diseases, pregnancy or lactation, treatment with other hypolipidemic drugs or with other dopamine agonists within 6 months preceding the study, concomitant treatment with angiotensin-converting enzyme inhibitors or sartans, concomitant treatment with nonsteroidal anti-inflammatory drugs, concomitant treatment with drugs known to interact with statins or cabergoline, and poor patient compliance.

After study approval by the Local Review Board, all participants provided signed informed consent to take part in the study. All procedures were conducted in full conformance with the principles of the Declaration of Helsinki.

### Study Design

For 14 weeks, all participants received atorvastatin (20 mg daily), while cabergoline in groups A and B was continued with the same dose. In addition to medical treatment, all patients were given standard weight-loss counseling and encouraged to follow a low-fat diet. Compliance with the medication regimen was evaluated by counting unused tablets at each study visit.

### Measurements

Anthropometric and blood pressure measurements were performed by standardized methods and trained medical personnel as previously described [16]. Carotid intima-media thickness (CIMT) was recorded on both sides between the carotid bulb origin and a point 15 mm proximal to the common carotid artery using high resolution B-mode ultrasound (Toshiba Aplio-500).

Peripheral blood samples for laboratory assays were obtained at approximately 8:00 a.m., following at least a 12-h overnight fasting at study entry and 14 weeks later. In groups A and B, prolactin, glucose homeostasis markers, plasma lipids, uric acids, and hsCRP were also assessed before implementation of cabergoline treatment. Blood was taken between days 2 and 5 of the menstrual cycle after the patient had been resting in the seated position for at least 30 min. The measurements were carried out in duplicate to minimize errors. Plasma concentrations of glucose, creatinine, total cholesterol, LDL cholesterol, HDL cholesterol, triglycerides, uric acid, albumin, and 25-hydroxyvitamin D were assayed using the multi-analyzers COBAS Integra 400 Plus and Cobas e 411 (Roche Diagnostics, Basel, Switzerland). Plasma levels of insulin, prolactin, total testosterone, estradiol, homocysteine, and sex hormone-binding globulin (SHBG) were detected by direct chemiluminescence using acridinium ester technology (ADVIA Centaur XP Immunoassay System; Siemens Healthcare Diagnostics, Munich, Germany). Circulating levels of hsCRP were measured using a chemiluminescent immunoassay (Immulite 2000XPi; Siemens Healthcare, Warsaw, Poland). Fibrinogen was assessed using the Clauss method (BCS XP autoanalyzer; Siemens Healthcare, Warsaw, Poland). The homeostasis model assessment of insulin resistance (HOMA-IR) was computed as follows: fasting insulin (μIU/mL) × fasting glucose (mg/dL)/405. The estimated glomerular filtration rate was calculated according to the Modification of Diet in Renal Disease equation. Free androgen index (FAI) was calculated as (total testosterone [nmol/L]/sex hormone-binding globulin [nmol/L]) ×100.

### Statistical Analysis

Because the raw data were skewed, a log transformation was applied to approximate normality. Comparisons between study populations and between percentage changes from baseline after adjustment for baseline values were carried out using one-way analysis of variance followed by the post hoc Bonferroni test. Values within the same group were compared using Student's paired *t* tests. The χ^2^ test was used for the comparison of categorical variables. Correlations were determined using Pearson's correlation coefficients. *p* values corrected for multiple testing below 0.05 were considered statistically significant.

## Results

**Fig. 2:**
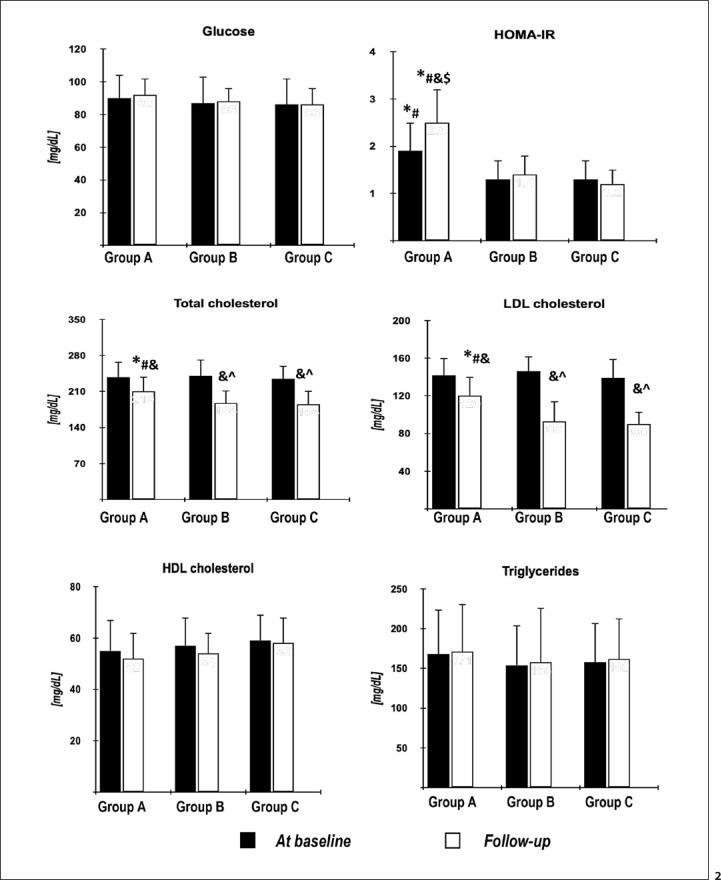
Baseline and follow-up values of glucose homeostasis markers and plasma lipids in the study population. ^^^statistically significant versus group A (*p* < 0.05); *statistically significant versus group B (*p* < 0.05); ^#^statistically significant versus group C (*p* < 0.05); ^&^statistically significant difference between posttreatment and baseline values within the same group (*p* < 0.05); ^$^percentage changes from baseline after adjustment for baseline values greater than in groups B and C (*p* < 0.05); ^^^percentage changes from baseline after adjustment for baseline values greater than in group A (*p* < 0.05).

**Fig. 3:**
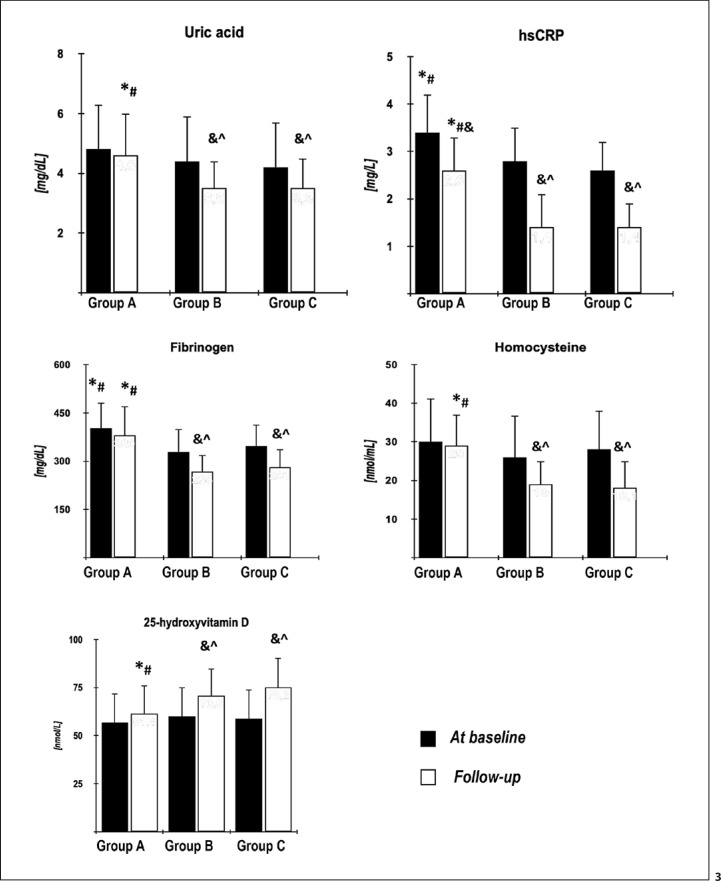
Baseline and follow-up values of the remaining risk factors in the study population. ^^^statistically significant versus group A (*p* < 0.05); *statistically significant versus group B (*p* < 0.05); ^#^statistically significant versus group C (*p* < 0.05); ^&^statistically significant difference between posttreatment and baseline values within the same group (*p* < 0.05); ^^^percentage changes from baseline after adjustment for baseline values more pronounced than in group A (*p* < 0.05).

At entry, there were no statistical differences between the groups in age, smoking, body mass index, waist circumference, blood pressure, CIMT, estradiol, glucose, lipids, uric acid, homocysteine, 25-hydroxyvitamin D, and the estimated glomerular filtration rate. Prolactin and testosterone levels and FAI were lower in group A than in groups B and C. HOMA-IR, hsCRP, and fibrinogen levels were higher in group A than in the remaining groups. There were no differences in the assessed variables between groups B and C (Tables 1, 2; Fig. 2, 3; online suppl. Table 1; for all online suppl. material, see www.karger.com/doi/10.1159/000527333). In group B, but not in group A, HOMA-1-IR, uric acid, and hsCRP were higher before initiation of cabergoline treatment than at baseline, while the opposite relationship was found for HDL cholesterol. There were no differences between both time points for glucose, total cholesterol, LDL cholesterol, and triglycerides (online suppl. Table 2).

Atorvastatin treatment was well tolerated and no patient dropped out because of adverse effects. However, one woman with hypoprolactinemia had follow-up prolactin levels within the reference, while another woman (from group C) had follow-up prolactin levels above the upper limit of normal. The results of both patients were not included in the statistical analysis. Cabergoline dose and treatment duration were similar in groups A and B [1.05 (0.40) *vs*. 0.83 (0.29) mg weekly (*p* = 0.12); 10 (2) *vs*. 11 (3) months (*p* = 0.35)].

There were no differences between baseline and follow-up values of body mass index, waist circumference, blood pressure, and CIMT (data not shown). Atorvastatin treatment was not associated with changes in prolactin, total testosterone, estradiol, glucose, HDL cholesterol, and triglyceride levels. The drug reduced total cholesterol, LDL cholesterol, and hsCRP levels in all study groups, but the reduction was greater in groups B and C than in group A. Only in groups B and C, atorvastatin decreased uric acid, fibrinogen, and homocysteine, as well as increased 25-hydroxyvitamin D. Only in group A, atorvastatin reduced FAI and increased HOMA-IR. Groups B and C were characterized by lower follow-up values of HOMA-IR, total and LDL cholesterol, uric acid, hsCRP, fibrinogen, and homocysteine and higher follow-up values of prolactin, testosterone, FAI, and 25-hydroxyvitamin D than group A (Table 2; Fig. 2, 3; online suppl. Table 1).

In group A, prolactin concentrations correlated with HOMA-IR (*r* = −0.442; *p* = 0.002), HDL cholesterol (*r* = 0.322; *p* = 0.035), uric acid (*r* = −0.378; *p* = 0.008), hsCRP (*r* = −0.501; *p* < 0.001), fibrinogen (*r* = −0.392; *p* = 0.007), testosterone (*r* = 0.420; *p* = 0.010), FAI (*r* = 0.460; *p* = 0.001), and CIMT (*r* = −0.298; *p* = 0.048). In this group, there were also correlations between baseline prolactin levels and the impact of atorvastatin on HOMA-IR (*r* = −0.355; *p* = 0.023), uric acid (*r* = 0.413; *p* = 0.008), hsCRP (*r* = 0.520; *p* < 0.001), fibrinogen (*r* = 0.470; *p* = 0.001), homocysteine (*r* = 0.362; *p* = 0.025), and 25-hydroxyvitamin D (*r* = 0.384; *p* = 0.010).

## Discussion

The results of the current study seem to be in line with previous observations suggesting that in patients in which hyperprolactinemia treatment leads to normal prolactin levels, cardiometabolic parameters have similar values as in untreated women with normal prolactin levels. The novel finding of the present study is that prolactin deficiency was characterized by impaired insulin sensitivity, low-grade systemic inflammation, and disturbed coagulation, as reflected by elevated values of HOMA-IR, hsCRP, and fibrinogen. They cannot be explained by a dose- or a time-dependent effect of cabergoline because both groups receiving this drug were well matched for the average dose and treatment duration. This means that using similar doses of cabergoline for a similar time period was associated with either the impaired cardiometabolic profile in individuals with prolactin deficiency or with the cardiometabolic profile indistinguishable from that observed in dopamine agonist-naïve apparently healthy young women in whom dopamine agonist doses were well chosen. Moreover, higher values of HOMA-IR and elevated concentrations of hsCRP and fibrinogen correlated with low prolactin levels but not with cabergoline dose and treatment duration. This finding suggests that dopaminergic agents may be effective in reducing the cardiometabolic risk associated with hyperprolactinemia only if they are administered in adequate but not excessive doses.

The unfavorable changes in glucose homeostasis markers in cabergoline-treated women with hypoprolactinemia were unexpected. Dopamine agonists are drugs that reduce plasma glucose levels, and a quick-release formulation of bromocriptine mesylate has been approved for the treatment of type 2 diabetes [17]. What is more, the impact of cabergoline on glucose homeostasis in individuals with elevated prolactin levels is stronger than that of bromocriptine [7]. The glucose-lowering effect of this group of agents is attributed to resetting of dopaminergic and sympathetic tone within the central nervous system, the consequence of which is the suppression of hepatic glucose production [17]. The results of the present study are the first to suggest that the impact of any dopamine agonist on glucose homeostasis depends on the prolactin status of patients. The lack of similar studies makes it difficult to draw any firm conclusions. The most likely explanation is that deterioration of glucose homeostasis associated with the presence of hypoprolactinemia may outweigh the benefits of the treatment with dopamine agonists itself.

Even more important finding of the present study is, however, the fact that the presence of prolactin deficiency was associated with impaired lipid-lowering and extra-lipid effects of atorvastatin. This observation cannot be interpreted as a result of late action of cabergoline or interactions (pharmacokinetic or pharmacodynamic) between cabergoline and statins because there were no differences between the atorvastatin actions in cabergoline-treated and dopamine agonist-naïve patients with prolactin levels within the reference range. Moreover, the degree of improvement in all biochemical variables assessed in the current study inversely correlated with prolactin levels in subjects with deficiency of this hormone. The unfavorable impact of prolactin deficiency on cardiometabolic effects was, however, less pronounced than that of monomeric hyperprolactinemia, completely abolishing the pleiotropic effects of statin therapy [14].

HMG-CoA reductase inhibitors, particularly the most potent ones (rosuvastatin and atorvastatin), may impair insulin sensitivity and increase the risk of new-onset diabetes [18]. The deteriorating impact of atorvastatin in the current study was observed only in women with low prolactin levels, was limited to an increase in HOMA-IR, as well as was observed despite the concomitant treatment with a strong dopamine agonist, known to exert a beneficial effect on glucose homeostasis [6, 7]. This finding is in line with the suggestions of other authors that some populations of patients may be more prone to the development of this complication. Untreated hypoprolactinemia may be one of these conditions, and the risk may be even greater if lactotroph hypofunction is not of iatrogenic origin, i.e., in organic disorders of the pituitary or of the hypothalamus.

Based on the obtained results, some comments may be drawn. First, it cannot be excluded that prolactin deficiency contributes to excess mortality induced by cardiovascular disease in females with hypopituitarism [13]. Second, dopaminergic agents should be administered with caution to high cardiovascular risk patients treated with statins and probably also other hypolipidemic agents. Furthermore, prolactin failure in some patient populations may require specific intervention (using lower doses of a dopamine agonist in drug-induced hypoprolactinemia or exogenous prolactin administration in individuals with organic lactotroph destruction). Finally, individuals poorly responding to HMG-CoA reductase inhibitors, treated with dopamine agonists, or with known disorders of the pituitary or surrounding tissues should be screened for the possible presence of prolactin failure.

We can only speculate on the molecular mechanisms explaining a similar effect on atorvastatin action of abnormally high and abnormally low prolactin levels. This similarity may reflect the fact that, depending on circumstances, prolactin may exert either pro- or anti-inflammatory effects [19], particularly may either activate [20] or inhibit the nuclear factor-κB pathway [21]. NF-κB is also an important signaling molecule, mediating anti-inflammatory, anticoagulant, and other pleiotropic effects of statins [22]. Alternatively, the effect of prolactin failure may be indirect and mediated by the impact on testosterone production or metabolism. In the current study, testosterone and FAI were lower in hypoprolactinemic than in normoprolactinemic women, while atorvastatin administration to women with prolactin failure led to a further decrease in FAI. The inhibitory effect of statin on testosterone production was observed previously in women with polycystic ovary syndrome [23] and congenital adrenal hyperplasia [24]. This explanation is also in line with the findings of other research teams showing that reduced androgen levels in women may predispose to cardiovascular disease [25], while severe atherosclerosis in postmenopausal women inversely correlates with free testosterone levels [26]. Finally, the inhibitory effect of prolactin failure may be associated with the impact on central mechanisms controlling immune response and hemostasis. This hypothesis is backed up by the facts that the hormone exerts a neuroprotective effect in the central nervous system, as well as that lactotroph hypofunction increases risk and severity of autoimmune encephalomyelitis in animals and multiple sclerosis in humans [27].

There are no universally accepted cutoff values defining hypoprolactinemia. In the current study, low and normal prolactin levels were defined based on the reference values established in our laboratory. The same diagnostic criteria for hypoprolactinemia (less than 5 ng/mL) were also used in our previous study [11] and in the study by Toledano et al. [28]. However, other research groups set different threshold levels. In the study by Diri et al. [29], prolactin deficiency in women with Sheehan's syndrome was diagnosed in individuals with basal prolactin concentration below 4.0 ng/mL and/or prolactin levels in the thyrotropin-releasing hormone stimulation test below 7.8 ng/mL. In turn, Sogawa et al. [30] diagnosed hypoprolactinemia in women with prolactin concentrations lower than 6.12 ng/mL. Because differences in the threshold levels were small, it is plausible that they do not have a significant effect on the obtained results.

We cannot exclude, however, that the impact of prolactin deficiency on cardiometabolic effects of atorvastatin may differ between men and women. Most conditions associated with low prolactin levels develop only (Sheehan's syndrome) or mainly (lymphocytic hypophysitis) in women, and women are treated with dopamine agonists much more often than men [9]. This explains why the prevalence of hypoprolactinemia in men is low and why it is difficult to collect a sufficient number of male subjects with prolactin deficiency. Although inclusion of only women helped us to obtain a more homogenous study population, the lack of a male group is undoubtedly a limitation of the current research.

Some other study limitations need to be mentioned. The most important limitation is the small study population, as well as short treatment duration. The study did not assess hard endpoints, and the obtained results cannot be easily translated to clinical outcomes. The study included only subjects with drug-induced hypoprolactinemia; it is not certain whether the impact of atorvastatin is similar in individuals with non-iatrogenic deficiency of this hormone. The study protocol does not make it possible to conclude whether the obtained results represent a class effect of statins or are related to specific properties of atorvastatin. Finally, because of exclusion criteria, the impact of subnormal prolactin levels on statin action in individuals with cardiovascular disease or diabetes requires further research.

## Conclusions

Compared with normoprolactinemic peers, women with drug-induced hypoprolactinemia were characterized by reduced insulin sensitivity and higher levels of hsCRP and fibrinogen. In individuals with subnormal prolactin levels, cardiometabolic effects of atorvastatin were relatively weakly expressed and inversely correlated with the degree of prolactin deficiency. These findings suggest that hypoprolactinemia may impair lipid-lowering and pleiotropic effects of statins, and those hypoprolactinemic patients treated with HMG-CoA reductase inhibitors may benefit from restoration of normal prolactin levels. Because of study limitations, larger-scale prospective studies assessing hard endpoints are required to confirm the obtained results and to clarify the clinical impact of hypoprolactinemia on cardiovascular disease management.

## Statement of Ethics

The study was conducted according to the guidelines of the Declaration of Helsinki and approved by the Institutional Review Board (the Bioethical Committee of the Medical University of Silesia [KNW/0022/KB/208/17]; May 16, 2017). Written informed consent to participate in the study was obtained from all participants.

## Conflict of Interest Statement

The authors have no conflicts of interest to declare.

## Funding Sources

This work did not receive any specific grant from funding agencies in the public, commercial, or not-for-profit sectors.

## Author Contributions

Robert Krysiak conceived the study, participated in its design, performed the statistical analysis, as well as drafted and edited the manuscript. Karolina Kowalcze conducted the literature search, carried out the assays, and performed the statistical analysis. Bogusław Okopień participated in the study design and coordination and provided critical input during manuscript preparations. All the authors read and approved the final manuscript.

## Data Availability Statement

All data generated or analyzed during this study are included in this manuscript and its online supplementary material. Further inquiries can be directed to the corresponding author.

## Supplementary Material

Supplementary data

Supplementary data

## Figures and Tables

**Table 1:** Baseline characteristics of the study population

	Group A^1^	Group B^2^	Group C^3^
Patients, *n*	11	15	25
Age, years; mean (SD)	32 (7)	31 (6)	30 (6)
Smokers, (%)/number of cigarettes a day, n; mean (SD)/duration of smoking, months, mean (SD)	27/10(6)/95 (40)	27/11 (7)/91(36)	38/10 (6)/88 (32)
Body mass index, kg/m^2^; mean (SD)	25.2 (4.5)	24.3 (4.0)	24.0 (3.5)
Waist circumference, cm; mean (SD)	80 (8)	77 (8)	76 (7)
Systolic blood pressure, mm Hg; mean (SD)	130 (16)	125 (17)	124 (15)
Diastolic blood pressure, mm Hg; mean (SD)	74 (7)	73 (6)	73 (6)
Intima-media thickness, mm; mean (SD)	0.65 (0.07)	0.61 (0.06)	0.61 (0.07)

SD, standard deviation.

1 Women with cabergoline-induced hypoprolactinemia.

2 Cabergoline-treated women with prolactin levels within the reference range.

3 Cabergoline-naïve women with prolactin levels within the reference range.

**Table 2:** Baseline and follow-up values of prolactin, total testosterone, the FAI, estradiol, and the estimated glomerular filtration rate in the study population

Variable	Group A^1^	Group B^2^	Group C^3^
Prolactin, ng/mL; mean (SD)			
At baseline	3.1 (1.3)^4^, ^5^	14.9 (5.8)	15.0 (5.2)
Follow-up	3.2 (1.4)^4^, ^5^	16.0 (5.7)	13.8 (5.0)
Total testosterone, nmol/L; mean (SD)			
At baseline	1.26 (0.35)^4^, ^5^	1.64 (0.39)	1.56 (0.38)
Follow-up	1.05 (0.29)^4^, ^5^	1.70 (0.41)	1.67 (0.40)
FAI, *n* (%); mean (SD)			
At baseline	2.10 (0.34)^4^, ^5^	2.69 (0.40)	2.76 (0.61)
Follow-up	1.82 (0.26)^4^, ^5^, ^6^, ^7^	2.78 (0.53)	2.90 (0.47)
Estradiol, pmol/L; mean (SD)			
At baseline	136 (60)	147 (55)	141 (56)
Follow-up	143 (58)	148 (48)	150 (50)
Estimated glomerular filtration rate, mL/min/1.73 m^2^; mean (SD)			
At baseline	96 (16)	93 (17)	98 (19)
Follow-up	94 (18)	96 (15)	98 (14)

SD, standard deviation.

1 Women with cabergoline-induced hypoprolactinemia.

2 Cabergoline-treated women with prolactin levels within the reference range.

3 Cabergoline-naïve women with prolactin levels within the reference range.

4 Statistically significant versus group B (*p* < 0.05).

5 Statistically significant versus group C (*p* < 0.05).

6 Statistically significant difference between posttreatment and baseline values within the same group (*p* < 0.05).

7 Percentage changes from baseline after adjustment for baseline values greater than in groups B and C (*p* < 0.05).
