# Supplementary material for: Cabergoline-Induced Hypoprolactinemia May Attenuate Cardiometabolic Effects of Atorvastatin: A Pilot Study
Source: Cardiology. 2022 Oct 4;147(5-6):497–506. doi: 10.1159/000527333 (PMC9808652; doi:10.1159/000527333)
Supplement: Supplementary file 1 — Supplementary data [file crd-0147-0497-s01.docx]

**Supplementary Table 1.** Baseline and follow-up values of glucose homeostasis markers, plasma lipids and the remaining cardiometabolic risk factors in the study population

| **Variable** | **Group A^1^** | **Group B^2^** | **Group C^3^** |
| --- | --- | --- | --- |
| **Prolactin [ng/mL; mean (SD)]**  *At baseline*  *Follow-up* | 3.1 (1.3)*^#^  3.2 (1.4)*^#^ | 14.9 (5.8)  16.0 (5.7) | 15.0 (5.2)  13.8 (5.0) |
| **Total testosterone** [nmol/L; mean (SD)]  *At baseline*  *Follow-up* | 1.26 (0.35)*^#^  1.05 (0.29)*^#^ | 1.64 (0.39)  1.70 (0.41) | 1.56 (0.38)  1.67 (0.40) |
| **Free androgen index** [%; mean (SD)]  *At baseline*  *Follow-up* | 2.10 (0.34)*^#^  1.82 (0.26)*^#&$^ | 2.69 (0.40)  2.78 (0.53) | 2.76 (0.61)  2.90 (0.47) |
| **Estradiol** [pmol/L; mean (SD)]  *At baseline*  *Follow-up* | 136 (60)  143 (58) | 147 (55)  148 (48) | 141 (56)  150 (50) |
| **Glucose** [mg/dL, mean (SD)]  *At baseline*  *Follow-up* | 90 (11)  92 (14) | 87 (12)  88 (15) | 86 (12)  86 (16) |
| **HOMA-IR** [mean (SD)]  *At baseline*  *Follow-up* | 1.9 (0.6)*^#^  2.5 (0.7)*^#&$^ | 1.3 (0.4)  1.4 (0.4) | 1.3 (0.4)  1.2 (0.3) |
| **Total cholesterol** [mg/dL; mean (SD)]  *At baseline*  *Follow-up* | 238 (30)  210 (29)*^#&^ | 240 (32)  188 (23)^&^^ | 235 (25)  185 (26)^&^^ |
| **LDL-cholesterol** [mg/dL; mean (SD)]  *At baseline*  *Follow-up* | 142 (18)  120 (20)*^#&^ | 146 (16)  93 (21)^&^^ | 139 (20)  90 (13)^&^^ |
| **HDL-cholesterol** [mg/dL; mean (SD)]  *At baseline*  *Follow-up* | 55 (12)  52 (10) | 57 (11)  54 (8) | 59 (10)  58 (10) |
| **Triglycerides** [mg/dL; mean (SD)]  *At baseline*  *Follow-up* | 168 (56)  171 (60) | 154 (50)  158 (68) | 158 (49)  162 (51) |
| **Uric acid** [mg/dL; mean (SD)]  *At baseline*  *Follow-up* | 4.8 (1.5)  4.6 (1.4)*^#^ | 4.4 (1.3)  3.5 (0.9)^&^^ | 4.2 (1.1)  3.5 (1.0)^&^^ |
| **hsCRP** [mg/L; mean (SD)]  *At baseline*  *Follow-up* | 3.4 (0.8)*^#^  2.6 (0.7)*^#&^ | 2.8 (0.7)  1.4 (0.7)^&^^ | 2.6 (0.6)  1.4 (0.5)^&^^ |
| **Fibrinogen** [mg/dL; mean (SD)]  *At baseline*  *Follow-up* | 402 (80)*^#^  380 (91)*^#^ | 329 (70)  268 (51)^&^^ | 346 (67)  281 (56)^&^^ |
| **Homocysteine** [nmol/mL; mean (SD)]  *At baseline*  *Follow-up* | 30.0 (11.2)  29.0 (8.0)*^#^ | 26.0 (10.8)  19.0 (5.9)^&^^ | 28.1 (10.0)  18.1 (6.8)^&^^ |
| **25-hydroxyvitamin D** [nmol/L; mean (SD)]  *At baseline*  *Follow-up* | 56.8 (15.0)  61.4 (14.6)*^#^ | 60.1 (12.5)  78.0 (14.0)^&^^ | 58.9 (11.6)  75.2 (15.3)^&^^ |
| **Estimated glomerular filtration rate**  [ml/min/1.73m^2^; mean (SD)]  *At baseline*  *Follow-up* | 96 (16)  94 (18) | 93 (17)  96 (15) | 98 (19)  98 (14) |

^1^women with cabergoline-induced hypoprolactinemia; ^2^cabergoline-treated women with prolactin levels within the reference range; ^3^cabergoline-naïve women with prolactin levels within the reference range

*statistically significant *vs.* group B (p<0.05)

^#^statistically significant *vs.* group C (p<0.05)

^&^statistically significant difference between post-treatment and baseline values within the same group (p<0.05)

^$^percentage changes from baseline after adjustment for baseline values greater than in groups B and C (p<0.05)

^^^percentage changes from baseline after adjustment for baseline values greater than in group A (p<0.05)

**Abbreviations:** hsCRP: high sensitivity C-reactive protein; HDL: high-density lipoprotein; HOMA-IR: homeostasis model assessment of insulin resistance; LDL: low-density lipoprotein; SD: standard deviation
